# Network-based repurposing identifies anti-alarmins as drug candidates to control severe lung inflammation in COVID-19

**DOI:** 10.1371/journal.pone.0254374

**Published:** 2021-07-22

**Authors:** Emiko Desvaux, Antoine Hamon, Sandra Hubert, Cheïma Boudjeniba, Bastien Chassagnol, Jack Swindle, Audrey Aussy, Laurence Laigle, Jessica Laplume, Perrine Soret, Pierre Jean-François, Isabelle Dupin-Roger, Mickaël Guedj, Philippe Moingeon

**Affiliations:** 1 Servier, Research and Development, Suresnes Cedex, France; 2 Lincoln, Research and Development, Boulogne-Billancourt Cedex, France; University of Maryland School of Medicine, UNITED STATES

## Abstract

While establishing worldwide collective immunity with anti SARS-CoV-2 vaccines, COVID-19 remains a major health issue with dramatic ensuing economic consequences. In the transition, repurposing existing drugs remains the fastest cost-effective approach to alleviate the burden on health services, most particularly by reducing the incidence of the acute respiratory distress syndrome associated with severe COVID-19. We undertook a computational repurposing approach to identify candidate therapeutic drugs to control progression towards severe airways inflammation during COVID-19. Molecular profiling data were obtained from public sources regarding SARS-CoV-2 infected epithelial or endothelial cells, immune dysregulations associated with severe COVID-19 and lung inflammation induced by other respiratory viruses. From these data, we generated a protein-protein interactome modeling the evolution of lung inflammation during COVID-19 from inception to an established cytokine release syndrome. This predictive model assembling severe COVID-19-related proteins supports a role for known contributors to the cytokine storm such as IL1β, IL6, TNFα, JAK2, but also less prominent actors such as IL17, IL23 and C5a. Importantly our analysis points out to alarmins such as TSLP, IL33, members of the S100 family and their receptors (ST2, RAGE) as targets of major therapeutic interest. By evaluating the network-based distances between severe COVID-19-related proteins and known drug targets, network computing identified drugs which could be repurposed to prevent or slow down progression towards severe airways inflammation. This analysis confirmed the interest of dexamethasone, JAK2 inhibitors, estrogens and further identified various drugs either available or in development interacting with the aforementioned targets. We most particularly recommend considering various inhibitors of alarmins or their receptors, currently receiving little attention in this indication, as candidate treatments for severe COVID-19.

## Introduction

Since the emergence of the new strain of Coronavirus SARS-CoV-2 in December 2019, the ongoing crisis associated with the COVID-19 disease has affected more than 170 million individuals worldwide, causing over 3.5 million deaths (World Health Organization Dashboard, June 1^st^, 2021), mainly as the consequence of an Acute Respiratory Distress Syndrome (ARDS). The pandemic is still progressing actively despite lockdown measures throughout the world, with the recent emergence of highly transmissible viral strains [1]. To date, the only proven medications for reducing either viral loads, hospitalization rates, invasive mechanical ventilation or patient mortality include corticosteroids such as dexamethasone, the antiviral remdesivir, the anti-IL6R tocilizumab as well as neutralizing monoclonal antibodies directed to the spike protein of the virus [2–5]. Many additional drugs have been tested, including the lopinavir antiviral, the anti-malarial hydroxychloroquine or IFNβ with as of today disappointing efficacy results [6].

Recently, several vaccines have been approved by regulatory authorities based on remarkable efficacy results, with evidence that they can protect against infection by eliciting high titers of neutralizing antibodies against the Spike protein of the SARS-CoV-2 virus [7]. Whereas such vaccines will very positively transform the course and gravity of the COVID-19 pandemic, a recent concern is whether they will be fully effective against emerging new variants of the virus bearing point mutations in the Spike protein [1]. Furthermore, the challenge of manufacturing and administering billions of vaccine doses in order to establish a protective herd immunity at a worldwide population level will not be met in a short time frame.

During the time needed to deploy preventive vaccines at such a scale, the repurposing of existing drugs is a valid solution to better address severe forms of COVID-19 and alleviate the burden on health services in a time and cost-effective manner. Previous repurposing strategies have been undertaken in the context of a limited understanding of COVID-19 pathogenesis, prompting to use related viruses such as SARS-CoV and MERS-CoV as proxies to model SARS-CoV-2 infection [8–13]. Several network computing studies have been successful to predict drug disease associations for repurposing in COVID-19. Many of those initial approaches were aiming to identify existing compounds to prevent viral infection by either targeting mechanisms involving the viral receptor ACE2 (angiotensin converting enzyme 2), the TMPRSS2 transmembrane protease serine 2, or clathrin-mediated endocytosis [14–16]. In the present repurposing study, we rather focused on drugs predicted to interfere with pro-inflammatory mediators identified by modelling immune dysregulations caused in the airways by SARS-CoV-2 infection.

Since a vast majority of patients infected with SARS-CoV-2 develop no or only mild symptoms, we reasoned that ideal candidate drugs to repurpose should rather inhibit severe airways inflammation in the course of the disease. Lung inflammation is the main cause requiring hospitalization in up to 20% of COVID-19 cases, with life threatening ARDS affecting 75% of COVID-19 patients transferred to intensive care units [17]. In this subset of patients with severe lung inflammation, persisting proinflammatory immune responses result in a cytokine release syndrome (CRS) linked to the activation of myeloid cells secreting cytokines such as IL1β, IL6 and TNFα [18–20].

Capitalizing on the most recent scientific insights on the pathophysiology of COVID-19, we undertook computational network analyses to integrate a wide variety of data sources encompassing extensive molecular profiling of SARS-CoV-2 infected epithelial or endothelial cells, genetic susceptibilities and immune dysregulations linked to severe COVID-19 as well as molecular mechanisms elicited during lung infection by other respiratory viruses. From this approach, a short list of COVID-19 disease-related proteins considered as potential therapeutic targets was established and used to computationally assess a topological proximity with drug targets within the comprehensive human protein-protein interactome [21, 22]. Herein, we report on the identification of candidate therapeutic targets, as well as drugs predicted to interact with some of those targets which could be repurposed to prevent or slow down severe lung inflammation during COVID-19.

## Materials and methods

### Sources of data on COVID-19 pathophysiology

To identify proteins related to lung inflammation in COVID-19, we selected relevant categories of data from the scientific literature (detailed in S1 Table), such as genes differentially expressed following SARS-CoV-2 infection of (i) primary normal human bronchial epithelial cells (*NHBE*) or of the ACE2-expressing lung-epithelial *Calu-3* cell line, (ii) endothelial cells or cells recovered from bronchoalveolar lavages or lung biopsies of patients with severe COVID-19 [23–25]. We also mined public data regarding immunological signatures obtained in the blood or in tissues of patients, distinguishing those with mild COVID-19 from others rather affected by severe forms of the disease [26–34]. We included as well information from previous studies on lung inflammation caused by other respiratory viruses (including asthma exacerbation), in light of an involvement of monocytes, macrophages, myeloid dendritic cells, innate lymphoid cells in those conditions similarly to COVID-19 [18, 35–38].

### Identification of disease-related proteins

COVID-19 disease-related proteins predicted to be involved in early lung inflammation and in the transition to the cytokine storm were identified following data mining from scientific publications listed in S1 Table. To establish molecular pathways dysregulated during lung inflammation due to COVID-19, we first used RNAseq data from *NHBE* (normal human bronchial epithelial) and *Calu-3* (human lung epithelial cancer) cells infected or not with SARS-CoV-2. These data were pre-treated by removing outlier samples whose total sum of counts was below 5 000 000. In order to filter out genes undistinguishable from background noise, we modelled gene expression after applying a log2(x + 1) transformation by a two component Gaussian mixture model, with a first peak corresponding to unexpressed genes, and the second peak to truly expressed genes. Numbers of genes pre and post-filtering were 17557 and 21797, respectively. We retrieved the parameters of the mixture distribution using function normalmixEM from mixtools package and determined that the 0.95 quantile for the noise distribution was 1.6. We subsequently removed all genes whose expression was below that threshold in more than 95% of samples. We performed a differential analysis (COVID versus mock) in each cell line using the *limma R* package and *eBayes* function (with mock group corresponding to healthy & no treatment patients). Disease signatures were then extracted by considering differentially expressed genes (DEG) as those with adjusted *p*-value below 0.05 with an absolute fold change superior to 1.3 (commonly used as a threshold for biological significance). Canonical pathway enrichment analyses were subsequently performed by using the Ingenuity Pathway Analysis (IPA) software.

### Network-based drug repurposing

Network-based drug repurposing relies on the hypothesis that the closer a target is to a group of disease related genes in the PPI network, the higher the chance of having a significant impact on the disease. Many approaches focus on the shortest path to determine proximity, with some variations in order to avoid hub protein bias [15, 39]. The latter bias occurs from certain proteins that have an extremely high degree in the network and thereby cause a highly dense graph. Other approaches take advantage of the diffusion process to define proximity [40] while considering all the topological features of the graph. Diffusion based metrics have a comparable advantage over shortest path distances when in highly dense graphs such as PPI graphs [41]. Other metrics distinct from shortest path and diffusion can be used such as such as largest connected component -based methods [42].

**Fig 1:**
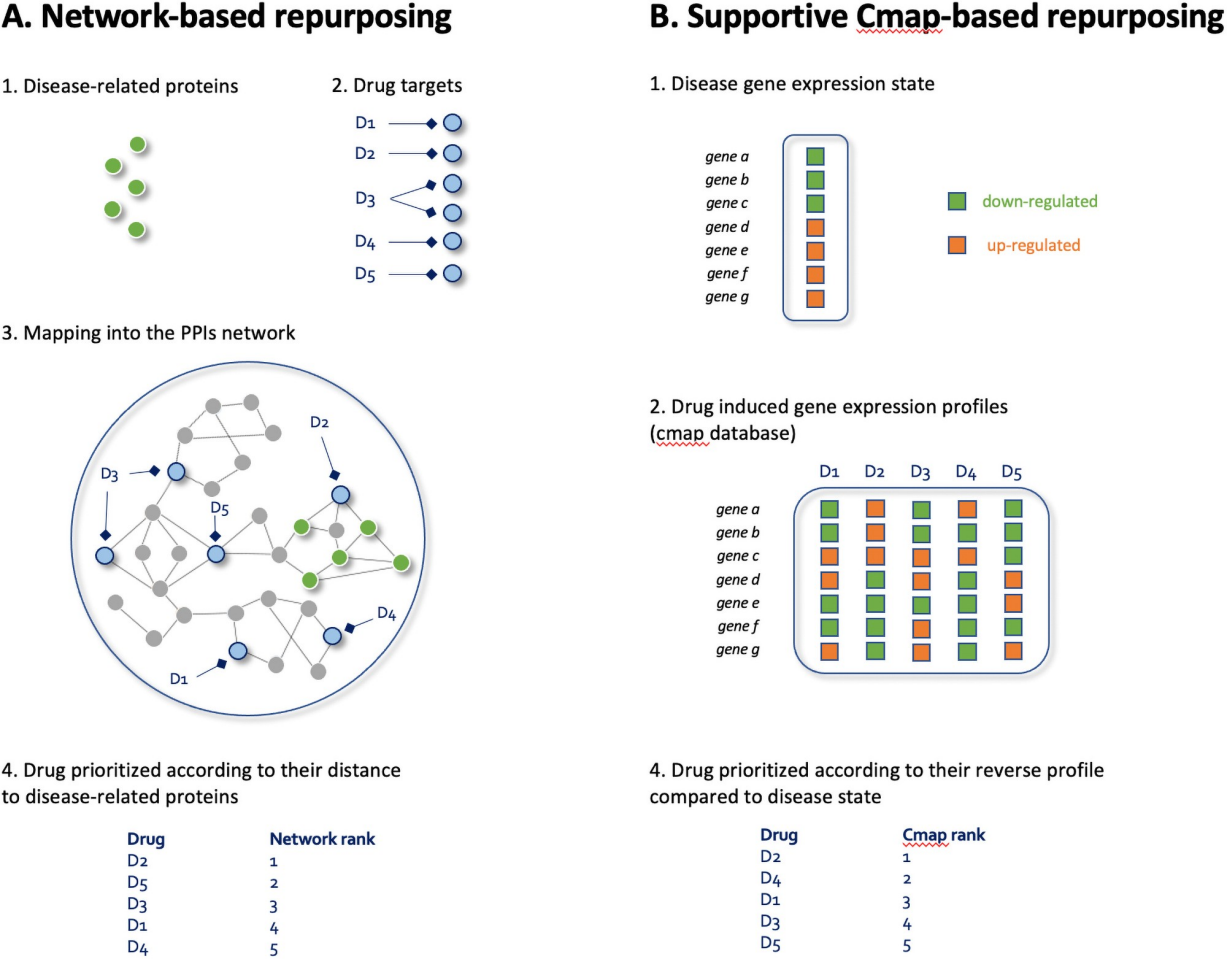
General principles of network and Cmap-based repurposing approaches. A) Network-based repurposing. Disease-related proteins and drug targets are mapped into a network of protein-protein-interactions (PPI). Drugs are prioritized according to their distance to disease-related proteins. B) Supportive Cmap-based repurposing. In those supportive analyses, disease-related as well as drug induced gene expression states are compared in order to identify drugs eliciting reverse profiles compared to those found in the disease.

Our computational repurposing approach (Fig 1A) takes advantage of the proximity between disease-related proteins and drug targets through an established network of protein-protein interactions (PPIs, referred to as an *interactome*). Drug-target links were gathered from the Therapeutic Target Database (TTD, version 7.1.01) and Drugbank [43, 44]. The PPIs network was derived from previous work by Cheng et al [45]. It was built from 15 different databases such as BioGRID and HPRD by compiling binary PPIs tested by high-throughput yeast-two-hybrid (Y2H) systems, kinase-substrate interactions from literature-derived low-throughput and high-throughput experiments, high-quality PPIs from three-dimensional (3D) protein structures, and signaling networks from literature-derived low-throughput experiments.

Relevance of drugs to the disease was assessed based on proximity of their targets to disease-related proteins according to two complementary metrics, namely a simple *topological distance* and a more advanced *diffusion-based distance*.

The *topological distance* (*d*_topo_) corresponds to the shortest path length in the PPIs network between the disease-related proteins and the drug targets, computed according to the following formula:

$$d_{\text{topo}}\left(P,T\right)=\frac{1}{\left\|T\right\|}\underset{t\in T}{\sum }\underset{p\in P}{\text{min}}\;SP\left(p,t\right)$$

With *P* the set of nodes corresponding to the disease-related proteins, *T* the set of nodes corresponding to the drug targets, and *SP*(*p*,*t*) the shortest path length between a node *p* of *P* and another node *t* of *T*. When calculating a topological distance, we generate a distribution from bootstrapping similar nodes defined by same degree in the graph. From the given distribution, we calculate a z-score (and p-value).

The *diffusion-based distance* (*d*_diff_) is computed based on the similarity of the impact on the network of perturbations starting from disease-related proteins on one side and drug targets on the other. The impact of a perturbation starting from a given node *n*_*i*_ on the network is assessed by use of a *diffusion* algorithm. Let (*n*_i_,*n*_j_) being a pair of nodes, then $\mathbb{P}\left(n_{i},n_{j}\right)$ represents the random walk-based probability that a perturbation starting from *n*_*i*_ reaches *n*_*j*_. It allows us to define a numerical vector *V (n*_*i*_*)* representing the impact perturbation of *n*_*i*_ on the whole interactome:

$$V\left(n_{i}\right)=\left[\mathbb{P}\left(n_{i},\;n_{1}\right),\;\mathbb{P}\left(n_{i},\;n_{2}\right),\;\ldots ,\;\mathbb{P}\left(n_{i},n_{n}\right)\right]$$

The similarity between two perturbations starting from *n*_*i*_ and *n*_*j*_ is then assessed by computing the Manhattan distance between *V* (*n*_*i*_) and *V* (*n*_*j*_). In order to extend this principle to the distance between sets of nodes, we derived the following formula:

$$d_{\text{diff}}\left(P,T\right)=\frac{1}{\left\|T\right\|}\underset{t\in T}{\sum }\underset{p\in P}{\text{min}}MD\left(p,t\right)$$

With *P* the set of nodes corresponding to the disease-related proteins, *T* the set of nodes corresponding to the drug targets, *p* one given node of *P*, *t* one given node of *T*, and *MD* (*p*,*t*) the Manhattan distance between *V*(*p*) and *V*(*t*). This diffusion-based distance was implemented via the DSD algorithm [46]. For each diffusion-based distance, we also calculate associated z-scores (and p-values). Note that DSD is by construction normally distributed. In order to prioritize drugs from this network-based repurposing approach, we defined a network rank resulting from the mean rank aggregation of *d*_topo_ and *d*_diff_. Given that we have p-values for both of our distance measures, we perform a Fisher’s combined probability test to obtain a unique combined p-value per drug. Using the DSD algorithm, we generated a computed distance matrix of 15 894 X 15 894 encompassing all proteins in our interactome.

### Cmap-based drug repurposing

We complemented the network-based approach by using Cmap as a supportive method (Fig 1B). Cmap identifies drugs inducing a reverse gene expression profile compared to the disease state using a method of similarity [47]. The Cmap database comprises human cancer cell lines either treated or not with chemical drugs, referred to as perturbagens. We used the *R* package *ccdata* which encompasses expression profiles for 1309 perturbagens over 13832 genes. Disease state was obtained from gene expression profiles induced in *NHBE* and *Calu-3* cells following infection by SARS-CoV-2. We compare expression profiles induced by disease state with those induced by perturbagens, using mainly the Pearson correlation between transcriptome values of the query signature and the perturbagen signature. A negative correlation score provides a potential therapeutic indication of the perturbagen. Cmap scores (the smaller the better) were first computed on both *NHBE* and C*alu-3* data and then averaged.

## Results and discussion

### Identification of COVID-19 disease-related proteins

**Fig 2:**
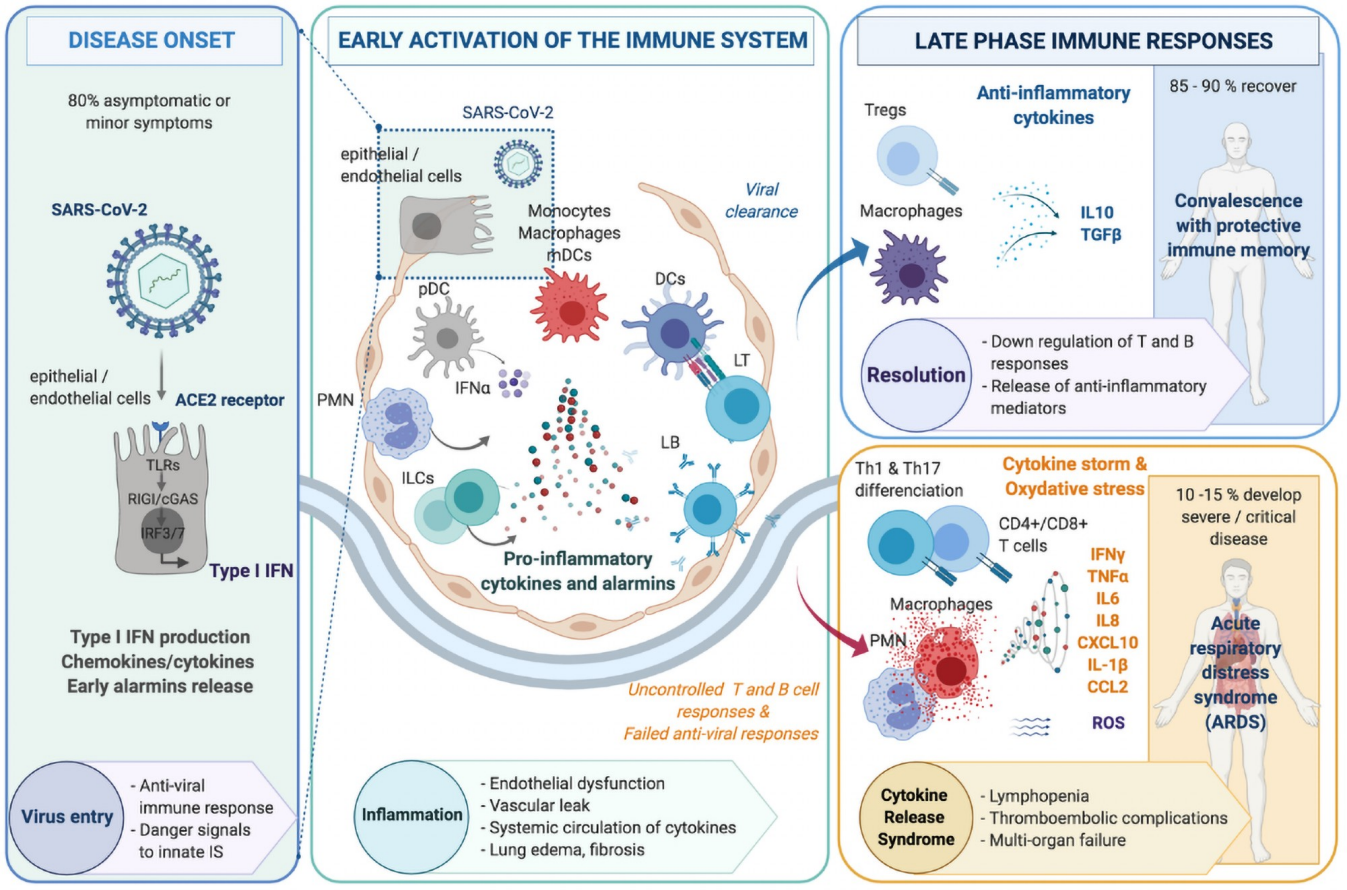
Three step progression towards severe COVID-19. The pathophysiology of COVID-19 in the airways encompasses schematically three successive steps, including **(i)** Disease onset following viral infection of alveolar epithelial or endothelial cells expressing the ACE2 receptor (left panel) leading to the activation of the innate immune system, with IFNα production by plasmacytoid dendritic cells (pDC). **(ii)** An early inflammatory phase within lung tissues where a cross-talk between infected epithelial/endothelial cells and innate immune cells such as monocytes, macrophages, myeloid dendritic cells (mDC) and innate lymphoid cells (ILCs) leads to a release of pro-inflammatory alarmins, cytokines and chemokines (center panel). This results in the activation of adaptive immunity, involving both CD4+ T cell help, CD8+ T cells cytotoxic for virally-infected cells as well as production of neutralizing antibodies against surface viral antigens. **(iii)** A late inflammatory phase with two potential outcomes: 85 to 90% of cases evolve towards resolution of inflammation with downregulation of T and B cell responses concomitant with the release of anti-inflammatory mediators (right upper panel); whereas 10 to 15% patients rather exhibit major tissue damage and severe acute respiratory distress syndrome (ARDS) caused by a deleterious uncontrolled inflammation linked with persisting T cell activation, excessive myeloid cell activation associated with a cytokine storm as well as oxidative stress (right lower panel).

Based on recent scientific advances, the pathophysiology of COVID-19 can be summarized as three sequential steps (Fig 2). We reasoned that treatments suitable to control severe COVID-19 should interfere with molecular pathways involved in the evolution from mild to severe lung inflammation (Fig 2, central panel), while preserving anti-viral protective immune mechanisms. We thus compiled a comprehensive list of genes differentially upregulated in *NHBE* and *Calu-3* human epithelial cells following SARS-CoV-2 infection, providing important quantitative information [23]. We cross-validated this list in comparison with molecular signatures reported at the level of endothelial cells, bronchoalveolar lavage cells or lung biopsies in other studies to be associated with severe COVID-19 or exposure to other respiratory viruses (S1 Table). The latter was further completed with deep immunophenotyping, RNA seq and cytokine profiling data related to dysregulated innate or adaptive immune responses in the blood or the lungs of patients with severe COVID-19. A compilation of the most relevant COVID-19 disease related-proteins thus obtained, together with data sources supporting their relevance to lung inflammation in COVID-19 are presented in S1 Table.

**Fig 3:**
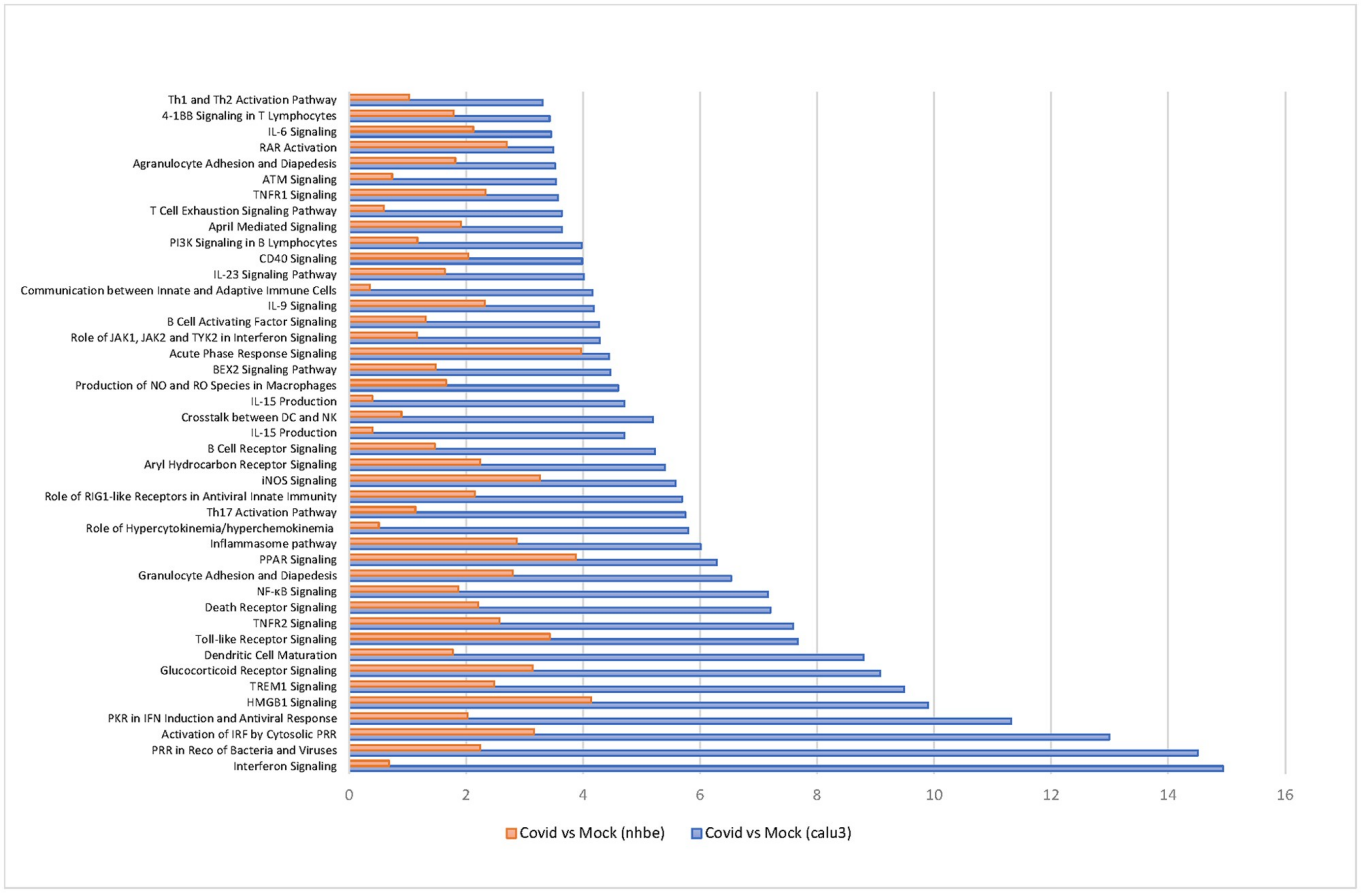
Pathway enrichment analysis from disease signatures (COVID-19 versus mock) in epithelial cell lines infected by SARS-CoV-2. The top 40 most significantly dysregulated immunological canonical pathways in either the Calu-3 (yellow) and NHBE (brown) infected cell lines are represented in a radar plot according to -log (*p*-value). Pathway enrichment *z*-scores, based on fold change direction, represent predicted up-regulation (green dots) or down-regulation (blue dots) for positive or negative values, respectively.

Ingenuity pathway analyses were then performed on this list, allowing to confirm that genes/proteins upregulated following SARS-CoV-2 infection in the airways belong to multiple well-known pro-inflammatory pathways (Fig 3, S2 Table). Further data interpretation led us to classify disease-related proteins in two distinct sets of highly represented proinflammatory mediators and cytokines termed *Alarmins* and *Cytokine storm*, respectively (S1 Table). Alarmins represent a family of immunomodulatory proteins acting as damage-associated molecular patterns provided by injured stromal cells to recruit and activate various innate immune cells such as monocytes, macrophages, innate lymphoid cells as well as myeloid dendritic cells. Multiple proteins belonging to this family (*i*.*e*. defensins, HMGB1, IL1α, IL25, IL33, TSLP, S100A4, S100A7, S100A8, S100A9, S100A12, S100B, S100P) as well as their receptors such as IL1R1, RAGE, ST2 were predicted by our model to be involved in the evolution towards severe lung inflammation in COVID-19.

Our study also draws attention on disease-related proteins linked to the cytokine storm occurring in severe forms of COVID-19. The latter includes proinflammatory cytokines produced by activated myeloid cells such as IL1β, IL6 and TNFα directly involved as a cause of the CRS observed in COVID-19 [18, 35, 36]. Other potential targets associated with the cytokine storm include various cytokines (*e*.*g*. IL1β, IFNγ, IL2, IL12, IL15, IL17, IL23, IL32), chemokines (*e*.*g*. CCL5, CCL20, CXCL5, CXCL10, CXCL11), as well as selected proinflammatory factors (*e*.*g*. JAK1, JAK2, C5a) (S1 Table) [19, 20, 26–28, 36, 48–50].

### Mapping into the interactome and identification of drug candidates for repurposing

**Fig 4:**
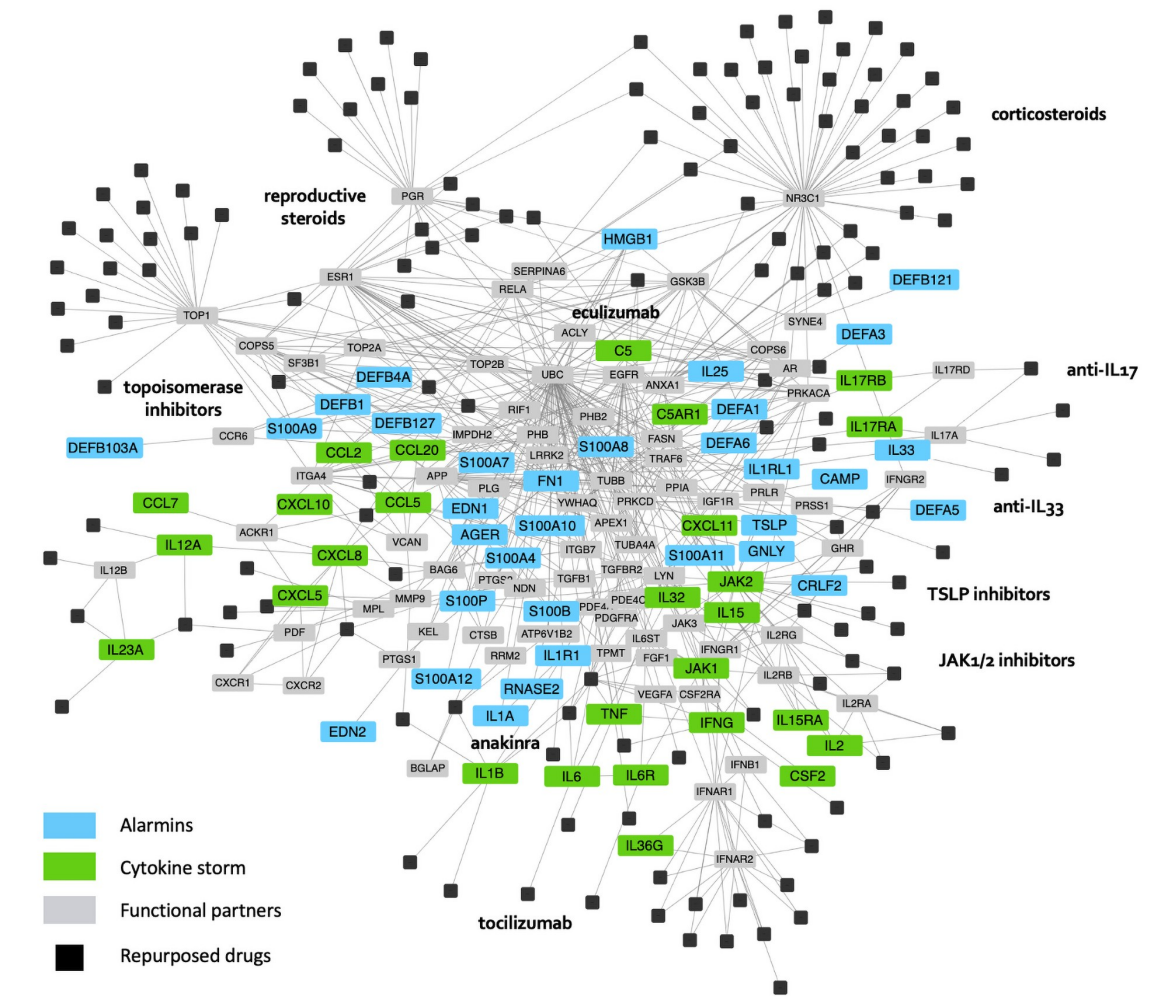
Druggable interactome of proteins contributing to lung inflammation in COVID-19. Extraction of the interactome encompassing proteins predicted to contribute to COVID-19 evolution towards a cytokine storm. Following SARS-CoV-2 infection of lung tissues and ensuing activation of innate and adaptive immune cells, different categories of proteins represent potential therapeutic targets to prevent or slow down lung inflammation associated with severe COVID-19. The latter include *Alarmins*, as well as cytokines, chemokines and selected proinflammatory factors associated with the *Cytokine storm*. For clarity, this figure only displays the disease related proteins (Alarmins & Cytokine storm) identified in our model, our top ranking repurposed drugs as well as some functional partners. The latter represent additional proteins needed in order to form a minimal principal component graph.

COVID-19 disease-related proteins were mapped in parallel with known drug targets into the human complete interactome made of 15894 proteins (including 951 known drug targets) and 213861 interactions (Fig 4). From this, 3092 drugs were ranked according to computational proximity of their targets to each of the alarmins and cytokine storm sets by using a network-based method (S3 Table). Both COVID-19-related proteins as well as some functionally-related proteins in the interactome (such as the NR3C1 glucocorticoid receptor or receptors for reproductive steroids) were identified as candidate therapeutic targets.

Table 1 provides a list of selected targets as well as drugs interacting with those targets predicted to be of interest in severe COVID-19. Specifically, several high-ranking drugs were identified to treat severe COVID-19, such as anti-IL1β, anti-IL6 and IL6R or anti-TNFα antibodies. Our model supports as well the interest of corticosteroids such as dexamethasone, broadly used currently to treat severe COVID-19 [2]. Other high-ranking candidates for repurposing identified in our study are JAK2 inhibitors, with drugs not yet approved such as momelotinib or gandotinib previously shown by structure-based virtual screening to interact with ACE2 and the SARS-CoV-2 main protease, but also baricitinib, as well as other JAK1/JAK2 inhibitors currently being evaluated in COVID-19 patients (Table 1). Interestingly, some network computing approaches aiming to repurpose drugs inhibiting cell infection by SARS-CoV-2 also concluded to the interest of blocking antibodies against IL1β, IL6 and TNFα as well as JAK inhibitors in treating COVID-19 patients, in agreement with the present study [15, 16]. In addition, we also identify several reproductive steroids (estrogens and progesterone) as interesting candidates for treating COVID-19 patients.

**Table 1:** Overview of main therapeutic targets and clinical-stage candidate drugs for repurposing in COVID-19- related lung inflammation.

Therapeutic targets [Disease-related genes]	Candidate drugs for repurposing [Company name]	Modalities	Marketed drugs: Yes/No	Clinical status in COVID-19 [Clinical trial ref]	Ref.
**Cytokine Release Syndrome: IL1β, IL6, TNFα and their receptors**	**Anti-IL1 β** Canakinumab [Novartis]	Antibody	Yes	Completed phase 2 in COVID-19 severe pneumonia [NCT04476706]. No impact on survival without the use of an invasive artificial respirator.	[4, 5, 18, 35, 56–58]
	**Anti-IL1 β** GLS1027 [GeneOne Life Science]	Small molecule	No	Recruitment planned for phase 2 in severe COVID-19 pneumonia [NCT04590547].	
	**Anti-IL6** Clazakizumab [CSL Limited]	Antibody	No	Ongoing phase 2 in life-threatening COVID-19 infection [NCT04343989].	
	**Anti-IL6** Olokizumab [R-Pharm]	Antibody	Yes	Completed phase 3 in acute respiratory distress syndrome [NCT04380519]. Results not yet available.	
	**Anti-IL6** Siltuximab [EUSA Pharma]	Antibody	Yes	Ongoing phase 3 in acute respiratory Distress Syndrome [NCT04616586].	
	**Anti-IL6** Sirukumab [Johnson & Johnson]	Antibody	No	Ongoing phase 2 in severe COVID-19 infection [NCT04380961].	
	**Anti-IL6R** Sarilumab [Sanofi]	Antibody	Yes	Completed phase 3 in severe or critical COVID-19 infection [NCT04327388], which did not meet its primary endpoint. Some improvement in survival when treating critically ill COVID-19 patients in association with dexamethasone.	
	**Anti-IL6R** Tocilizumab [Roche]	Antibody	Yes	Several trials completed in severe COVID-19 showing only limited efficacy [NCT04381936]. Some improvement in survival when treating critically ill COVID-19 patients in association with dexamethasone.	
	**Anti-TNFα** Infliximab [Johnson & Johnson]	Antibody	Yes	Ongoing phase 3 in COVID-19 [NCT04593940].	
	**Anti-TNFα** Adalimumab, [AbbVie]	Antibody	Yes	Ongoing phase 3 in mild to moderate COVID-19 [NCT04705844].	
	**TNF-α inhibitor** XPro-1595 [INmune Bio]	Peptide	No	Ongoing phase 2 in pulmonary complications of COVID-19 [NCT04370236].	
	**Anti-TNFα** Etanercept [Amgen]	Fusion protein	Yes	No evaluation yet in COVID-19.	
**Glucocorticoid receptor NR3C1**	**Corticosteroids** Dexamethasone [Mylan], Hydrocortisone [Sanofi-Aventis], Prednisolone [Mylan]	Small agonist molecules	Yes	Positive results obtained in the RECOVERY phase 3 study [NCT04381936], confirmed by a WHO-sponsored meta-analysis of 7 randomized clinical trials, collectively providing evidence for a reduced mortality of critically ill patients.	[2, 59]
				Dexamethasone is broadly used as a treatment for severe COVID-19.	
**JAK1, JAK2**	**JAK1/JAK2 inhibitor** Baricitinib [Eli Lily]	Small molecule	Yes	Ongoing phase 2 in moderate pneumonia [NCT04358614]. Recent evidence that Baricitinib can inhibit viral entry by clathrin-mediated endocytosis.	[60, 61]
	**JAK/JAK2 inhibitor** Ruxolitinib [Novartis]	Small molecule	Yes	Ongoing phase 2 in severe COVID-19 pneumonia [NCT04359290].	
	**JAK2 inhibitor** Jaktinib [Suzhou Zelgen Biopharmaceutical]	Small molecule	No	Completed phase 2 in severe and acute exacerbation of COVID-19 pneumonia [ChiCTR2000030170].*	
	**JAK2 inhibitor** Pacritinib [CTI BioPharma]	Small molecule	No	Ongoing phase 3 in severe COVID-19 [NCT04404361].	
	**JAK2 inhibitor** TD-0903 [Theravance Biopharma]	Small molecule	No	Ongoing phase 2 in symptomatic acute lung injury associated with COVID-19 [NCT04402866].	
**Reproductive steroids:** Estrogens, progesterone and their receptors	**Receptor agonists** Ethinylestradiol + Norelgestromin [Johnson & Johnson]	Small molecules	Yes	Planned phase 2 in non-severe COVID-19 patients [NCT04539626].	[63]
**Cytokines: IL2, IL15, IL17**	**IL2Rβ superagonist** Bempegaldesleukin [Nektar]	Recomb protein	No	Ongoing phase 1b in mild COVID-19 [NCT04646044].	[52]
	**IL15 super agonist** ALT803 [Altor Biosciences]	Recomb protein	No	Planned phase 1 study in mild to moderate COVID-19.	
	**Anti-IL17** Secukinumab [Novartis]	Antibody	Yes	Ongoing phase 2 in mild and severe COVID 19 [NCT04403243].	
	**Anti-IL17, -IL17R, -IL23**	Antibodies	Yes	No evaluation yet in COVID-19. Anti IL17 [Ixekizumab, Eli Lilly], anti IL17R [Brodalumab, Astra Zeneca/ Amgen], anti IL23 [Ustekinumab, Johnson & Johnson; Tildrakizumab, Merck] antibodies are commercialized as treatments for inflammatory diseases.	
**C5, C5aR**	**Anti C5** Eculizumab [Alexion]	Antibody	Yes	Proof-of-concept evidence suggesting that eculizumab provides some benefit in severe COVID-19. Ongoing phase 2 in moderate, severe or critical COVID-19 pneumonia [NCT04346797].	[53, 64–66]
	**Anti C5aR** Avdoralimab [Innate Pharma]	Antibody	No	Ongoing phase 2 in severe COVID-19 pneumonia [NCT04371367].	
**Alarmins and their receptors: IL1α, TSLP, IL33**	**IL1R1 antagonist** Anakinra [Sobi]	Peptide	Yes	Completed phase 2 in severe COVID-19 [NCT04366232]. Results not yet available.	[71, 75, 77]
	**Anti-IL33R [ST2]** AMG282-Astegolimab [Genentech]	Antibody	No	Ongoing phase 2 in severe COVID-19 Pneumonia [NCT04386616].	
	**TSLP inhibitor** HY-209- NuSepin [Shaperon] agonist for G protein-coupled TGR5 receptor	Small molecule	No	Ongoing phase 2 in COVID-19 pneumonia [NCT04565379].	
	**Anti IL25, -IL33, -TSLP**	Antibodies	No	No evaluation yet in COVID-19. Anti IL25 [ABM-125, Abeome], Anti-IL33 [REGN3500, Regeneron] and anti TSLP [Teepelumab, Amgen] are in clinical evaluation as treatments for asthma or atopic dermatitis.	
	**Anti S100A4, -S100A7,—S100P**	Antibodies	No	No evaluation yet in COVID-19. Antibodies in preclinical development in cancer or autoimmune diseases by Cancer Res Technol and Lykera Biomed.	
**Thrombopoietin receptor**	**Receptor agonist** Romiplostim [Amgen]	Peptibody [peptide agonist fused to Fc IgG1]	Yes	Case study documenting platelet recovery following treatment by Romiplostim of a pediatric patient with thrombocytopenia due to COVID-19.	[54]

All clinical trial information are available in Clinical trials gov: https://www.clinicaltrials.gov/ or* in Chinese clinical trial Registry: http://www.chictr.org.cn/.

Whereas the previous targets and some of the drugs directed to them could be expected from the current state of knowledge, our modeling study provided as well interesting hypotheses regarding other therapeutic options receiving less attention as of today. For example, drugs interacting with alarmins were also strongly suggested to be useful in COVID-19. To our knowledge, only three clinical studies have been initiated in COVID-19 with anti-alarmins, despite the availability of multiple additional drug candidates in this class (Table 1). Noteworthy, since Alarmins of the S100 family activate Toll-like receptors such as TLR2 and TLR4, a therapeutic option might be to target specific TLRs downstream of alarmins. Indeed, several TLR‐antagonists are currently undergoing clinical evaluation in order to restore immune‐homeostasis in patients with COVID‐19 [51].

Similarly, anti-IL17 antibodies rank very high in our repurposing analysis, suggesting that inhibitory drugs directed to this well-known pro-inflammatory cytokine as well as the functionally related IL23 cytokine or their receptors should be further investigated in COVID-19, with only one ongoing clinical trial in COVID-19 as of today [52]. In addition, the C5 complement inhibitor eculizumab is also predicted to represent an interesting treatment option, in agreement with recent evidence that the C5a-C5aR axis contributes to severe lung inflammation in COVID-19 patients [53]. As a strong chemoattractant, C5a provides in parallel to alarmins a link between innate and adaptive immune responses during severe COVID-19.

The thrombopoietin receptor appears as well to be a valid therapeutic target for agonists in light of the high incidence of thrombocytopenia associated with COVID-19 infection [54]. Rather unexpectedly, Topoisomerase 1 inhibitors, currently used as cytotoxic drugs in oncology, were also identified as of potential interest in COVID-19, with as of today only preclinical evidence that they can inhibit SARS-CoV-2 inflammation and death in animal models [55].

### Supportive Cmap-based for drug repurposing

Given the rather limited set of transcriptomics data available and the small Cmap coverage for repurposable drugs (*i*.*e*. only 17% of molecules in our drug database, with none of the biologics), results were taken as supportive in the present study. Among the top network-based drugs proposed for repurposing, only 2 corticosteroids (betamethasone and hydrocortisone) were confirmed to elicit a reversed gene expression profile (Cmap score < -0.3) when compared to the disease gene expression state.

## Conclusion

This study was designed to identify existing drugs which could be repurposed in a short time frame as a treatment for severe forms of COVID-19. We reasoned that such drugs should target those molecular pathways involved in the transition from mild lung inflammation caused by viral infection up to the cytokine storm associated with advanced stages of the disease (Fig 2, central and right lower panels). To this aim, using multiple sources of molecular profiling data from the literature relevant to distinguish mild from severe forms of the disease at the level of tissues and immune cells, we established a model of lung inflammation associated with COVID-19 in the form of an interactome of disease-related proteins. Combined with pharmacological knowledge of drug targets, this interactome allowed us to identify existing compounds which could be made available to patients in a short time frame.

Our network computational analyses identified several candidate therapeutic targets and corresponding drugs to repurpose which were confirmatory of existing knowledge (Table 1). This includes for example therapeutic antibodies interfering with either IL1β, IL6, TNFα or their receptors directly contributing to the CRS associated with severe COVID-19. Various inhibitory antibodies directed to these targets have already been evaluated in COVID-19 patients, such as anti-IL1^®^ (canakinumab), anti-IL6R (tocilizumab, sarilumab) or anti-TNFα (infliximab, adalimumab) antibodies [4, 56]. Overall, these drugs yielded conflicting efficacy results, likely explained by evidence that such anti-cytokine treatments are rather effective if administered to patients before they develop advanced COVID-19 [57]. Nonetheless, a recent study evaluating the anti-IL6R antibodies tocilizumab and sarilumab demonstrated some improvement in survival when treating critically ill COVID-19 patients, even more so when these drugs were associated with dexamethasone [4, 5, 58]. Corticosteroids, are also predicted by the present study to be useful in severe COVID-19, in agreement with positive results previously obtained in multiple randomized clinical trials, eventually leading to a broad use of dexamethasone as a treatment for severe COVID-19 [2, 59]. JAK1 and JAK2 inhibitors came out also as interesting candidates for repurposing, with several inhibitors being actively tested in COVID-19 patients [60]. In this therapeutic class, the JAK1/JAK2 inhibitor baricitinib is currently raising most of the interest in light of recent evidence that it interferes with virus entry mediated by clathrin-associated endocytosis (Table 1) [61]. We also identified drugs interfering with reproductive steroids or their receptors as valid candidates for repurposing. This observation makes sense in light of the strong bias towards males among patients with severe COVID-19, perhaps explained in part by the upregulation by androgens of the expression of the SARS CoV-2 receptor [62]. In contrast estrogens and progesterone are rather considered to be protective in light of their anti-inflammatory properties as well as their capacity to promote proliferation and repair of respiratory epithelial cells [63]. On this basis, treatment with estrogens are being considered in patients with mild COVID-19 (Table 1).

Perhaps more interestingly, our repurposing study sheds light on other therapeutic classes which as of today receive insufficient attention as potential treatments for severe COVID-19. We predict that inhibitors of the well-known IL17 and IL23 proinflammatory cytokines (or their receptors) could be useful in COVID-19, with to our knowledge a single clinical trial evaluating as of today the anti-IL17 antibody secukinumab in COVID-19 [52]. Multiple monoclonal antibodies blocking those cytokines have been registered as treatments for other inflammatory diseases, which thus could be promptly repurposed in COVID-19 (Table 1). Similarly, the C5 complement inhibitor eculizumab was also identified to represent a valid therapeutic option, in agreement with recent evidence that the C5a-C5aR axis promotes severe lung inflammation in COVID-19 patients by mediating recruitment and activation of pro-inflammatory myeloid cells [53, 64]. Only proof of concept studies have been conducted so far in human with eculizumab, suggesting that this antibody may provide some benefit in severe COVID-19 [65, 66], with a confirmatory trial ongoing in a larger cohort of patients. Noteworthy, another clinical study has been recently initiated to evaluate as well in this indication the anti C5a receptor antibody avdoralimab (Table 1). Also, approaches combining JAK1/2 inhibitors with blockade of C5a with eculizumab are being considered as a treatment of severe pulmonary damage in COVID-19 patients [67]. Moreover, drugs such as romiplostim acting as an agonist for the thrombopoietin receptor are also predicted to be useful to treat COVID-19-associated thrombocytopenia, in agreement with a recent case study documenting platelet recovery following treatment with this drug of a COVID-19 pediatric patient [54].

The most significant outcome of our repurposing study is the prediction that several members of the alarmin family such as defensins, HMBG1, IL1α, IL25, IL33, TSLP, S100A4, S100A7, S100A8, S100A9, S100A12, S100B, S100P likely contribute to lung inflammation during COVID-19 (Fig 4) [68–70]. The role of each individual alarmin in this regard remains to be investigated, with presumably some of them (*e*.*g*. IL25, TSLP) rather contributing to the initial recruitment of myeloid cells and innate lymphoid cells following epithelial or endothelial cell infection, whereas others (IL33, S100 members) are likely being involved in later stages of lung inflammation culminating in the cytokine storm. The later assumption is consistent with recent observations that some alarmins can stimulate the production of both IL1β, IL6 and TNFα as well as multiple other proinflammatory cytokines and chemokines [71]. Furthermore, blood levels of IL1α, calprotectin (a heterodimer made of S100A8 and S100A9), S100A12, S100B and HGBM1 appear to correlate with COVID-19 severity [72–76] (S1 Table). Also, IL33 has been recently proposed to play a broad role in the pathophysiology of COVID-19 pneumonia by dampening both the antiviral interferon response as well as regulatory T cells, while promoting thrombosis and activating pro-inflammatory type 2 innate lymphoid cells and γδ T cells [77]. To our knowledge, only few clinical studies are being conducted as of today in COVID-19 with a TSLP inhibitor or with blocking antibodies directed to receptors for IL1α or IL33 (i.e. ST2), whereas multiple additional blocking monoclonal antibodies directed to IL25, IL33 or TSLP are well under clinical evaluation to treat severe forms of asthma or atopic dermatitis [62, 69]. Furthermore, various inhibitors of the S100 family of proteins currently in preclinical development may represent promising drug candidates for the future (Table 1). We thus recommend considering existing anti-alarmins therapies to treat severe COVID-19, most particularly in the context of the converging rationale from this computational study as well as recent wet-lab evidence that this important class of proteins conveying proinflammatory signals plays a critical role in the pathophysiology of severe COVID-19. Lastly, this first model of severe lung inflammation in COVID-19 should be updated as new data are generated to better distinguish at an early stage patients with a high risk of evolving towards severe lung inflammation from those who will only develop mild forms of the disease.

## Supporting information

S1 Table Candidate COVID-19 related disease genes. (PDF)

S2 Table Pathways enrichment analysis. (PDF)

S3 Table Drug repurposing. (XLSX)
