# Supplementary material for: Network-based repurposing identifies anti-alarmins as drug candidates to control severe lung inflammation in COVID-19
Source: PLoS One. 2021 Jul 22;16(7):e0254374. doi: 10.1371/journal.pone.0254374 (PMC8297899; doi:10.1371/journal.pone.0254374)
Supplement: S1 Table — (PDF) [file pone.0254374.s001.pdf]

Supplementary Table 1: Candidate COVID-19 related disease genes

Gene Symbol	Gene ID	Gene expression on NHBE cells after SARS-CoV-2 infection (GSE147507)		Gene expression on Calu-3 cell line after SARS-CoV-2 infection (GSE147507)		Relevance of COVID-19 related proteins based on additional data sources: molecular profiling data of human lung tissues infected by SARS-CoV-2 or other respiratory viruses, deep-immunophenotyping/ cytokine profiling data in the blood or lungs of patients with severe COVID-19
		Log Fc	pValue	Log Fc	pValue	
**Cytokine Release Syndrome (CRS)**						
**C5**	727	NS	NS	NS	NS	- Elevated levels of C5a in bronchoalveolar liquid fluid (BALF) of COVID-19 patients with acute respiratory distress syndrome (ARDS) (Carvelli et al.)[1]<br>- High plasma levels of soluble C5b9 in COVID-19 patients (Peffault de Latour et al.)[2]<br>- Interest of targeting proinflammatory C5a in COVID-19 (Merad et al.)[3]
**C5aR1**	728	NS	NS	NS	NS	C5aR1+ cells detected in neutrophils and monocytes from lung samples of COVID-19 patients with ARDS (Carvelli et al.)[1]
**CCL2 (MCP-1)**	6347	NA	NA	2,27199792	0,00033869	- CCL2 gene expression detected in BALF of COVID-19 patients with ARDS (Carvelli et al.)[1]<br>- High expression of CCL2 in single-cell RNA sequenced BALF macrophages from COVID-19 patients (Liao et al.)[4]<br>- Elevated levels of circulating CCL2 associated with COVID-19 severity (Hadjadj et al.)[5]
**CCL20**	6364	2,80512267	0,00644687	3,72792364	0,0014312	Upregulated gene expression in SARS-CoV infected in human peripheral blood mononuclear cells (PBMCs) (Ng et al.)[6]
**CCL5**	6352	NS	NS	4,0079621	0,00071506	
**CCL7 (MCP-3)**	6354	NS	NS	NS	NS	- Increased plasma levels of CCL7 in COVID-19 patients associated with disease severity (Vabret et al.)[7]<br>- High expression of CCL7 in single-cell RNA sequenced BALF macrophages from COVID-19 patients (Liao et al.)[4]
**CSF2 (GM-CSF)**	1437	1,12015328	0,00070066	4,43508382	7,3065E-17	Increased frequency of GM-CSF+IFN-γ+ T cells and CD14+CD16+GM-CSF+ monocytes in blood from COVID-19 patients (Vabret et al.)[7]
**CXCL10 (IP10)**	3627	NS	NS	4,6211212	0,00072944	- Increased plasma levels of CXCL10 in COVID-19 patients associated with disease severity (Vabret et al., Laing et al.)[7,8]<br>- High expression of CXCL10 in single-cell RNA sequenced BALF macrophages from COVID-19 patients (Liao et al.)[4]<br>- Increased CXCL10 gene expression in lung biopsies from COVID-19 patients (Ackermann et al.)[9]
**CXCL11**	6373	NS	NS	4,53540857	0,0007608	High expression of CXCL11 on single-cell RNA sequenced BALF macrophages from COVID-19 patients (Liao et al.)[4]
**CXCL5**	6374	1,79511867	3,0178E-6	1,03717803	0,00855694	- CXCL5 contributes to SARS-CoV-2 induced lung inflammation in a hACE2 transgenic mouse model (Laing et al.)[8]<br>- Implication of CXCL5-mediated neutrophil recruitment in viral-induced asthma exacerbation (Sokulsky et al.)[10]<br>- Top hub gene from transcriptome network analysis of SARS-CoV-2 infected human cell (Fagone et al.)[11]
**CXCL8 (IL8)**	3576	NA	NA	NA	NA	- Increased plasma levels of CXCL8 in COVID-19 patients associated with disease severity (Vabret et al., Laing et al.)[7,8]<br>- CXCL8 detected in BALF of COVID-19 patients with ARDS (Carvelli et al.)[1]<br>- Increased CXCL8 gene expression in lung biopsies from COVID-19 patients (Ackermann et al.)[9]<br>- Top hub gene from transcriptome network analysis of SARS-CoV-2 infected human cell (Fagone et al.)[11]
**IFNγ**	3458	NA	NA	NA	NA	- Increased plasma levels of IFNγ in COVID-19 patients (Vabret et al.)[7]<br>- Interest of targeting IFNγ in COVID-19 (Merad et al., Ye et al.)[3,12]<br>- IFNγ implication in coronavirus recognition and immune response, in relationship with obesity, age, and risk of severe COVID-19 (Brodin)[13]
**IL12A**	3592	NS	NS	2,06155735	7,6616E-06	IL12A implication in coronavirus recognition and immune response, in relationship with obesity, age, and risk of severe COVID-19 (Brodin)[13]
**IL15**	3600	NS	NS	1,37007695	0,00492881	

Gene Symbol	Gene ID	Gene expression on NHBE cells after SARS-CoV-2 infection (GSE147507)		Gene expression on Calu-3 cell line after SARS-CoV-2 infection (GSE147507)		Relevance of COVID-19 related proteins based on additional data sources: molecular profiling data of human lung tissues infected by SARS-CoV-2 or other respiratory viruses, deep-immunophenotyping/ cytokine profiling data in the blood or lungs of patients with severe COVID-19
		Log Fc	pValue	Log Fc	pValue	
**IL15RA**	3601	NS	NS	1,9033827	0,00013765	
**IL1β**	3553	1,03550935	0,00911132	3,91985098	4,991E-12	- IL1β detected in BALF of COVID-19 patients with ARDS (Carvelli et al.)[1]<br>- High expression of IL1β in single-cell RNA sequenced BALF macrophages from COVID-19 patients (Liao et al.)[4]<br>- Increased blood levels of CD14+ IL1β+ monocytes in COVID-19 patients (Wen et al.)[14]<br>- Top hub gene from transcriptome network analysis of SARS-CoV-2 infected human cell (Fagone et al.)[11]<br>- IL1β implication in coronavirus recognition and immune response, in relationship with obesity, age, and risk of severe COVID-19 (Brodin)[13]
**IL2**	3558	NA	NA	NA	NA	Increased plasma levels of IL2 in COVID-19 patients (Vabret et al.)[7]
**IL23A**	51561	NS	NS	1,21854059	0,00120562	
**IL32**	9235	1,73869606	0,00014965	1,20922853	0,0134375	
**IL36G**	56300	2,16695495	5,883E-05	NS	NS	
**IL6**	3569	2,00320976	0,00194694	5,11939899	1,5515E-09	- Increased plasma levels of IL6 in COVID-19 patients associated with disease severity (Vabret et al., Laing et al., Hadjadj et al.)[5,7,8]<br>- IL6 detected in BALF of COVID-19 patients with ARDS (Carvelli et al.)[1]<br>- High expression of IL6 in single-cell RNA sequenced BALF macrophages from COVID-19 patients (Liao et al.)[4]<br>- Increased IL6 gene expression in lung biopsies from COVID-19 patients (Ackermann et al.)[9]<br>- Top hub gene from transcriptome network analysis of SARS-CoV-2 infected human cell (Fagone et al.)[11]<br>- Interest of targeting IL6 in COVID-19 (Merad et al., Ye et al., Moore et al., Rica et al.)[3,12,15,16]
**JAK1**		0,56164939	0,00984357	NS	NS	Interest of targeting JAK-STAT pathway in COVID-19 (Luo et al.)[17]
**JAK2**		NS	NS	1,45997096	0,00011929	Interest of targeting JAK-STAT pathway in COVID-19 (Luo et al.)[17]
**TNFα**	7124	NS	NS	4,77158416	1,827E-07	- TNFα detected in BALF of COVID-19 patients with ARDS (Carvelli et al.)[1]<br>- High expression of TNFα in single-cell RNA sequenced BALF macrophages from COVID-19 patients (Liao et al.)[4]<br>- Elevated TNFα serum concentration associated with critical COVID-19 (Leisman et al., Hadjadj et al.)[5,18]<br>- TNFα implication in coronavirus recognition and immune response, in relationship with obesity, age, and risk of severe COVID-19 (Brodin)[13]<br>- Interest of targeting TNFα in COVID-19 (Ye et al.)[12]
**IL17RA**	23765	NS	NS	NS	NS	Increased blood levels of IL17 in COVID-19 patients associated with lung injury (Pacha et al.)[19]
**Alarmins**						
**AGER (RAGE)**	177	NS	NS	NS	NS	Low sRAGE levels in elderly patients with lung involvement (Yalcine Kehribar et al.)[20]
**CAMP**	820	NS	NS	NS	NS	Role of Vitamin D in the regulation of CAMP gene expression in the course of COVID-19 disease (Roth et al.)[21]
**Defensin α**	Group ID 502	NA	NA	NA	NA	Downregulation of α-Defensin gene expression following SARS-CoV-2 infection (Idris et al.)[22]
**Defensin β**	Group ID 503	NA	NA	NA	NA	Downregulation of β-Defensin gene expression following SARS-CoV-2 infection (Idris et al.)[22]
**EDN1**	1906	NS	NS	1,86633662	0,00981865	
**EDN2**	1907	NS	NS	2,01436393	1,389E-05	
**FN1**		1,25633714	0,00806437	NS	NS	Increased FN1 gene expression in lung biopsies from COVID-19 patients (Ackermann et al.)[9]
**GNLY**	10578	NS	NS	NS	NS	Elevated CD4+ and CD8+ effector T cells expressing GNLY are associated with convalescence in moderate COVID-19 patients (Zhang et al.)[23]
**HMGB1**	3146	NS	NS	-0,7123577	0,00335352	Elevated serum levels of HMGB1 correlates with inferior clinical outcomes in COVID-19 patients (Chen et al.)[24]
**IL1A**	3552	NS	NS	4,95457015	2,0084E-12	Top hub gene from transcriptome network analysis of SARS-CoV-2 infected human cell (Fagone et al.)[11]
**IL1R1**	3554	0,50439323	0,05023721	NS	NS	Elevated serum levels of IL1R1 in COVID-19 patients (Choreño-Parra et al.)[25]

Gene Symbol	Gene ID	Gene expression on NHBE cells after SARS-CoV-2 infection (GSE147507)		Gene expression on Calu-3 cell line after SARS-CoV-2 infection (GSE147507)		Relevance of COVID-19 related proteins based on additional data sources: molecular profiling data of human lung tissues infected by SARS-CoV-2 or other respiratory viruses, deep-immunophenotyping/ cytokine profiling data in the blood or lungs of patients with severe COVID-19
		**Log Fc**	**pValue**	**Log Fc**	**pValue**	
**IL25**	64806	NA	NA	NA	NA	- Increased production of IL25 during lung inflammation associated with allergic asthma (Atamas et al.)[26]<br>- Interest of targeting IL25 in COVID-19 (Rica et al.)[16]
**IL33**	90865	NS	NS	NS	NS	- High serum IL33 levels correlates with adverse COVID-19 outcome (Burke et al., Ye et al., Zizzo et al.)[12,27,28]<br>- Increased production of IL33 during lung inflammation associated with allergic asthma (Atamas et al.)[26]<br>- Interest of targeting IL33 in COVID-19 (Rica et al.)[16]
**S100A4**	6275	NS	NS	NS	NS	S100A4+ T cells detected in the blood of COVID-19 patients (Zhang et al.)[23]
**S100A7**	6278	NS	NS	NS	NS	Top hub gene from transcriptome network analysis of SARS-CoV-2 infected human cell (Fagone et al.)[11]
**S100A7A**	338324	0,62524042	2,019E-07	NS	NS	
**S100A8**	6279	NS	NS	NS	NS	- Elevated gene expression levels of S100A8 mRNA and protein level in lung samples from fatal COVID-19 cases (Wu et al.)[29]<br>- Elevated serum levels of S100A8/A9 are correlated with inferior clinical outcomes in COVID-19 patients (Chen et al.)[24]<br>- Single-cell landscape of bronchoalveolar immune cells (Liao et al.)[4]<br>- Elevated plasma levels in severe COVID-19 patients (Silvin et al.)[30]<br>- Top hub gene from transcriptome network analysis of SARS-CoV-2 infected human cell (Fagone et al.)[11]
**S100A9**	6280	NS	NS	NS	NS	- Elevated gene expression levels of S100A9 mRNA and protein level in lung samples from fatal cases of COVID-19 (Wu et al.)[29]<br>- Elevated serum levels of S100A8/A9 are correlated with inferior clinical outcomes in COVID-19 patients (Chen et al.)[24]<br>- Elevated plasma levels in severe COVID-19 patients (Silvin et al.)[30]<br>- Top hub gene from transcriptome network analysis of SARS-CoV-2 infected human cell (Fagone et al.)[11]
**S100A10**	6281	NS	NS	-0,4801333	0,08343353	Elevated anti- Annexin A2 antibodies associated with high mortality in COVID-19 patients (Zuniga et al.)[31]
**S100A11**	6282	0,4543227	0,0858988	NS	NS	Elevated gene expression levels of S100A11 mRNA and protein level in lung samples in fatal COVID-19 cases (Wu et al.)[29]
**S100A12**	6283	0,43317817	0,06620747	NS	NS	- Increased gene expression levels of S100A12 mRNA and protein level in lung samples in fatal COVID-19 cases (Wu et al.)[29]<br>- Single-cell RNASeq detects S100A12+ neutrophils in the blood of patients with severe COVID-19 (Combes et al.)[32]<br>- Plasma levels of S100A12 elevated in severe COVID-19 patients (Arunachalam et al.)[33]<br>- Top hub gene from transcriptome network analysis of SARS-CoV-2 infected human cell (Fagone et al.)[11]
**S100B**	6285	NA	NA	NA	NA	Detection of serum S100B associated with disease severity (Aceti et al.)[34]
**S100P**	6286	NS	NS	1,47488119	0,02103115	Elevated gene expression of S100P mRNA and protein in lung samples in fatal cases (Wu et al.) [29]
**ST2**	9173	NA	NA	NA	NA	- Elevated serum levels of ST2 in patients with COVID-19 (Zeng et al.)[35]<br>- Interest of targeting ST2 based on increased expression in COVID-19 (Zizzo et al.)[28]
**TSLP**	85480	NS	NS	NS	NS	- Elevated serum levels of TSLP in COVID-19 patients (Choreño-Parra et al.)[25]<br>- Increased production of TSLP during lung inflammation associated with allergic asthma (Atamas et al.)[26]
**CRLF2 (TSLPR)**	14281	NA	NA	NA	NA	Elevated serum levels in COVID-19 patients (Choreño-Parra et al.)[25]

NA: *Not available*; NS: *Not significant*

References:

1 Carvelli J, Demaria O, Vély F, *et al.* Association of COVID-19 inflammation with activation of the C5a–C5aR1 axis. *Nature* 2020; **588**: 146–50.

2 Peffault de Latour R, Bergeron A, Lengline E, *et al.* Complement C5 inhibition in patients with COVID-19 - a promising target? *Haematologica* 2020; **105**: 2847–50.

3 Merad M, Martin JC. Pathological inflammation in patients with COVID-19: a key role for monocytes and macrophages. *Nature Reviews Immunology* 2020; **20**: 355–62.

4 Liao M, Liu Y, Yuan J, *et al.* Single-cell landscape of bronchoalveolar immune cells in patients with COVID-19. *Nature Medicine* 2020; **26**: 842–4.

5 Hadjadj J, Yatim N, Barnabei L, *et al.* Impaired type I interferon activity and exacerbated inflammatory responses in severe Covid-19 patients. *medRxiv* 2020; : 2020.04.19.20068015.

6 Ng LFP, Hibberd ML, Ooi E-E, *et al.* A human in vitro model system for investigating genome-wide host responses to SARS coronavirus infection. *BMC Infect Dis* 2004; **4**: 34.

7 Vabret N, Britton GJ, Gruber C, *et al.* Immunology of COVID-19: Current State of the Science. *Immunity* 2020; **52**: 910–41.

8 Laing AG, Lorenc A, del Molino del Barrio I, *et al.* A dynamic COVID-19 immune signature includes associations with poor prognosis. *Nature Medicine* 2020; **26**: 1623–35.

9 Ackermann M, Verleden SE, Kuehnel M, *et al.* Pulmonary Vascular Endothelialitis, Thrombosis, and Angiogenesis in Covid-19. *New England Journal of Medicine* 2020; **383**: 120–8.

10 Sokulsky LA, Garcia-Netto K, Nguyen TH, *et al.* A Critical Role for the CXCL3/CXCL5/CXCR2 Neutrophilic Chemotactic Axis in the Regulation of Type 2 Responses in a Model of Rhinoviral-Induced Asthma Exacerbation. *The Journal of Immunology* 2020; **205**: 2468–78.

11 Fagone P, Ciurleo R, Lombardo SD, *et al.* Transcriptional landscape of SARS-CoV-2 infection dismantles pathogenic pathways activated by the virus, proposes unique sex-specific differences and predicts tailored therapeutic strategies. *Autoimmunity Reviews* 2020; **19**: 102571.

12 Ye Q, Wang B, Mao J. The pathogenesis and treatment of the `Cytokine Storm' in COVID-19. *J Infect* 2020; **80**: 607–13.

13 Brodin P. Immune determinants of COVID-19 disease presentation and severity. *Nature Medicine* 2021; **27**: 28–33.

14 Wen W, Su W, Tang H, *et al.* Immune cell profiling of COVID-19 patients in the recovery stage by single-cell sequencing. *Cell Discovery* 2020; **6**: 1–18.

15 Moore JB, June CH. Cytokine release syndrome in severe COVID-19. *Science* 2020; **368**: 473–4.

16 de la Rica R, Borges M, Gonzalez-Freire M. COVID-19: In the Eye of the Cytokine Storm. *Front Immunol* 2020; **11**. DOI:10.3389/fimmu.2020.558898.

17 Luo W, Li Y-X, Jiang L-J, Chen Q, Wang T, Ye D-W. Targeting JAK-STAT Signaling to Control Cytokine Release Syndrome in COVID-19. *Trends in Pharmacological Sciences* 2020; **41**: 531–43.

18 Leisman DE, Ronner L, Pinotti R, *et al.* Cytokine elevation in severe and critical COVID-19: a rapid systematic review, meta-analysis, and comparison with other inflammatory syndromes. *The Lancet Respiratory Medicine* 2020; **8**: 1233–44.

19 Pacha O, Sallman MA, Evans SE. COVID-19: a case for inhibiting IL-17? *Nature reviews Immunology* 2020; **20**: 345–6.

20 Yalcin Kehribar D, Cihangiroglu M, Sehmen E, *et al.* The receptor for advanced glycation end product (RAGE) pathway in COVID-19. *Biomarkers* 2021; : 1–5.

21 Roth A, Lütke S, Meinberger D, *et al.* LL-37 fights SARS-CoV-2: The Vitamin D-Inducible Peptide LL-37 Inhibits Binding of SARS-CoV-2 Spike Protein to its Cellular Receptor Angiotensin Converting Enzyme 2 In Vitro. *bioRxiv* 2020; : 2020.12.02.408153.

22 Idris MM, Banu S, Siva AB, Nagaraj R. Downregulation of Defensin genes in SARS-CoV-2 infection. *medRxiv* 2020; : 2020.09.21.20195537.

23 Zhang J-Y, Wang X-M, Xing X, *et al.* Single-cell landscape of immunological responses in patients with COVID-19. *Nature Immunology* 2020; **21**: 1107–18.

24 Chen L, Long X, Xu Q, *et al.* Elevated serum levels of S100A8/A9 and HMGB1 at hospital admission are correlated with inferior clinical outcomes in COVID-19 patients. *Cellular & Molecular Immunology* 2020; **17**: 992–4.

25 Choreño-Parra JA, Jiménez-Álvarez LA, Cruz-Lagunas A, *et al.* Clinical and immunological factors that distinguish COVID-19 from pandemic influenza A(H1N1). *medRxiv* 2020; : 2020.08.10.20170761.

26 Atamas SP, Chapoval SP, Keegan AD. Cytokines in chronic respiratory diseases. *F1000 Biol Rep* 2013; **5**. DOI:10.3410/B5-3.

27 Burke H, Freeman A, Cellura DC, *et al.* Inflammatory phenotyping predicts clinical outcome in COVID-19. *Respiratory Research* 2020; **21**: 245.

28 Zizzo G, Cohen PL. Imperfect storm: is interleukin-33 the Achilles heel of COVID-19? *The Lancet Rheumatology* 2020; **2**: e779–90.

29 Wu M, Chen Y, Xia H, *et al.* Transcriptional and proteomic insights into the host response in fatal COVID-19 cases. *PNAS* 2020; **117**: 28336–43.

30 Silvin A, Chapuis N, Dunsmore G, *et al.* Elevated Calprotectin and Abnormal Myeloid Cell Subsets Discriminate Severe from Mild COVID-19. *Cell* 2020; **182**: 1401-1418.e18.

31 Zuniga M, Gomes C, Carsons SE, *et al.* Autoimmunity to the Lung Protective Phospholipid-Binding Protein Annexin A2 Predicts Mortality Among Hospitalized COVID-19 Patients. *medRxiv* 2021; : 2020.12.28.20248807.

32 Combes AJ, Courau T, Kuhn NF, *et al.* Global absence and targeting of protective immune states in severe COVID-19. *Nature* 2021; : 1–10.

33 Arunachalam PS, Wimmers F, Mok CKP, *et al.* Systems biological assessment of immunity to mild versus severe COVID-19 infection in humans. *Science* 2020; **369**: 1210–20.

34 A A, Lm M, E S, *et al.* Serum S100B protein as a marker of severity in Covid-19 patients. *Sci Rep* 2020; **10**: 18665–18665.

35 Zeng Z, Hong X-Y, Li Y, *et al.* Serum-soluble ST2 as a novel biomarker reflecting inflammatory status and illness severity in patients with COVID-19. *Biomarkers in Medicine* 2020; **14**: 1619–29.
