# Supplementary material for: Network-based repurposing identifies anti-alarmins as drug candidates to control severe lung inflammation in COVID-19
Source: PLoS One. 2021 Jul 22;16(7):e0254374. doi: 10.1371/journal.pone.0254374 (PMC8297899; doi:10.1371/journal.pone.0254374)
Supplement: S2 Table — (PDF) [file pone.0254374.s002.pdf]

Supplementary table 2: Pathways enrichment analysis

Pathways	Covid vs Mock (*Calu-3*)		Covid vs Mock (*NHBE*)	
	**-log(p-value)**	**z-score**	**-log(p-value)**	**z-score**
Interferon Signaling	14,9357719	4,123	0,683442248	0
PRR in Reco of Bacteria and Viruses	14,50864765	4,472	2,24189323	3
Activation of IRF by Cytosolic PRR	12,99480525	1,528	3,157269106	1,732
PKR in IFN Induction and Antiviral Response	11,31942565	3,8	2,027834347	2,84
HMGB1 Signaling	9,892816978	4,69	4,136325824	3,411
TREM1 Signaling	9,487339799	4,243	2,480392326	2,714
Glucocorticoid Receptor Signaling	9,076600929	0	3,141536633	0
Dendritic Cell Maturation	8,795978612	5,292	1,772605702	2,982
Toll-like Receptor Signaling	7,673581383	2,53	3,432181182	1,265
TNFR2 Signaling	7,593047721	1,667	2,565941826	1,633
Death Receptor Signaling	7,20278698	2,183	2,208166042	1,387
NF-κB Signaling	7,160638145	3	1,868981762	1,789
Granulocyte Adhesion and Diapedesis	6,529496585	0	2,79474373	0
PPAR Signaling	6,286538017	-2,828	3,879057447	-3,3
Inflammasome pathway	6,006448638	2,828	2,867468029	1,633
Role of Hypercytokinemia/hyperchemokinemia in the Pathogenesis of Influenza	5,79636691	0	0,509261684	0
Th17 Activation Pathway	5,752172021	3,5	1,133602884	3,162
Role of RIG1-like Receptors in Antiviral Innate Immunity	5,688755259	2,333	2,150794101	2,449
iNOS Signaling	5,584343342	3,162	3,267745421	2,121
Aryl Hydrocarbon Receptor Signaling	5,399895352	1,807	2,242282309	1,069
B Cell Receptor Signaling	5,224189356	3,545	1,463860724	2,524
IL-15 Production	4,710149251	3,638	0,402876847	2,333
Crosstalk between DC and NK	5,195791833	3,742	0,89461228	1,89
IL-15 Production	4,710149251	3,638	0,402876847	2,333
Production of NO and RO Species in Macrophages	4,607415684	3,71	1,658383408	2,236
BEX2 Signaling Pathway	4,469391464	0,277	1,482649183	1,265
Acute Phase Response Signaling	4,442869286	3,153	3,96473143	3,71
Role of JAK1, JAK2 and TYK2 in Interferon Signaling	4,282891999	0	1,166798588	0
B Cell Activating Factor Signaling	4,270401843	2,646	1,309580617	2,236
IL-9 Signaling	4,183126507	2,121	2,31853725	1,89
Communication between Innate and Adaptive Immune Cells	4,165170081	0	0,356106281	0
IL-23 Signaling Pathway	4,015157259	2,333	1,636391835	1,89
CD40 Signaling	3,984220892	0,632	2,033289628	1,897
PI3K Signaling in B Lymphocytes	3,973919273	3,357	1,16765933	2,496
April Mediated Signaling	3,637119196	1,414	1,910321188	1,89
T Cell Exhaustion Signaling Pathway	3,633300389	3,3	0,591510638	1,897
TNFR1 Signaling	3,568386692	1,414	2,32816913	2,121
ATM Signaling	3,535346727	-0,632	0,735966675	1,134
Agranulocyte Adhesion and Diapedesis	3,521694216	0	1,815185695	0
RAR Activation	3,492150953	0	2,698918044	0

**Pathways**	**Covid vs Mock (*Calu-3*)**		**Covid vs Mock (*NHBE*)**	
	**-log(p-value)**	**z-score**	**-log(p-value)**	**z-score**
IL-6 Signaling	3,451654986	3,873	2,121144973	3,5
4-1BB Signaling in T Lymphocytes	3,427157488	2,236	1,790275559	2,236
Th1 and Th2 Activation Pathway	3,310325046	0	1,022728542	0
